# Dose- and time-dependent effects of collagenase clostridium histolyticum injection on transverse carpal ligament elastic modulus and thickness *in vitro*

**DOI:** 10.1371/journal.pone.0277187

**Published:** 2022-12-01

**Authors:** Jocelyn L. Hawk, Sohail R. Daulat, David S. Margolis, Zong-Ming Li

**Affiliations:** Hand Research Laboratory, Departments of Orthopaedic Surgery and Biomedical Engineering, University of Arizona, Tucson, AZ, United States of America; Nanjing University of Science and Technology, CHINA

## Abstract

A potential treatment for carpal tunnel syndrome is to biochemically alter the mechanical properties of the transverse carpal ligament (TCL) through Collagenase Clostridium Histolyticum (CCH) injection. The purpose of this study was to determine the time- and dose-dependent effects of CCH injection on TCL elastic modulus and thickness. Nine TCLs were dissected from cadaveric hands for this study. CCH doses of 50U, 100U, 150U, 200U, and 250U were injected into five points on the TCL, respectively. B-mode and shear wave elastography images were taken of each injection point using robot-assisted ultrasound imaging immediately after injection, as well as 2, 4, 6, 8, and 24 hours after injection. TCL thickness and mean shear wave speed were measured for each CCH dose at each time point. CCH doses of 200U and 250U decreased shear wave speed by 18.70% and 30.01% (p<0.05), respectively, after 24 hours. CCH doses of 150U, 200U, and 250U decreased TCL thickness by 7.28%, 10.97%, and 14.92%, respectively, after 24 hours (p<0.05). Our findings suggest that CCH injection may be effective in degrading TCL tissue, with higher doses of CCH resulting in greater tissue degradation up to 24 hours after injection.

## Introduction

The transverse carpal ligament (TCL) is a band of tissue constituted by collagen fibers types I and III that forms the volar boundary of the carpal tunnel [1]. Important biomechanical functions of the TCL include serving as the origin site for the thenar and hypothenar muscles and acting as a pulley for the flexor tendons [2]. The TCL is relevant to carpal tunnel syndrome, one of the most common peripheral compression neuropathies [3, 4], due to its volar restriction of the median nerve. During wrist flexion and finger motion, the flexor tendons and median nerve move volarly and are compressed against the TCL [5, 6]. Carpal tunnel syndrome has also been associated with greater TCL stiffness and greater thickness in the central region of the TCL [7].

Existing interventions for mild carpal tunnel syndrome include wrist splinting and corticosteroid injections, but these treatments generally provide short-term relief and are not effective in more severe cases [8, 9]. The most common surgical procedure for carpal tunnel syndrome is carpal tunnel release, in which the TCL is transected to increase carpal tunnel space, decompressing the median nerve [10]. However, complete division of the TCL can disrupt the important biomechanical functions of the TCL and result in side effects, such as pillar pain and hand weakness [11–13]. A possible alternative to complete division of the TCL is to biochemically decrease TCL stiffness. Stiffness is dependent on elastic modulus and thickness, and lower TCL stiffness could allow for palmar bowing of the carpal arch, increasing carpal tunnel space. In addition to thickness directly affecting stiffness, a thinner TCL may increase carpal tunnel space by decreasing the bulk of boundary.

Collagenases are enzymes that degrade collagen fibers, and Collagenase Clostridium Histolyticum (CCH) is composed of clostridial type I and type II collagenases isolated from Clostridium histolyticum [14]. CCH injection has previously been used to treat Dupuytren’s contracture, a condition characterized by formation of collagen cords in the hand, causing finger contractures and impaired hand function [15–17]. CCH injection as a treatment for Dupuytren’s contracture has become increasingly used due to shorter recovery time and reduced complication rates when compared to surgical intervention [18, 19]. CCH injection to the TCL could be similarly used to break down collagen fibers in the TCL, potentially decreasing its stiffness.

TCL thickness and elastic modulus can be observed through ultrasound imaging. B-mode ultrasound imaging has been previously used for reliable measurements of the TCL, including thickness [20]. Shear wave elastography provides information on a tissue’s elasticity by propagating acoustic shear waves through the tissue, and shear wave speed is related to elastic modulus [21, 22]. In order to decrease TCL stiffness without disrupting the anatomy of the TCL, determining an optimal dose of CCH to deliver to the TCL is needed. Injecting sub-optimal CCH would not decrease TCL stiffness enough to allow the TCL to palmarly bow to decompress the median nerve. Injecting excessive CCH may rupture the TCL, disrupting its biomechanical functions and possibly affecting nearby structures. The objective of this study was to assess the time- and dose-dependent effects of CCH injection on the shear wave speed and thickness of the TCL *in vitro* using B-mode ultrasound imaging and shear wave elastography. We hypothesized that TCL degradation would increase with time for any dose of injection, and TCL shear wave speed and thickness would be negatively correlated with injected dose.

## Materials and methods

### Specimen preparation

**Fig 1:**
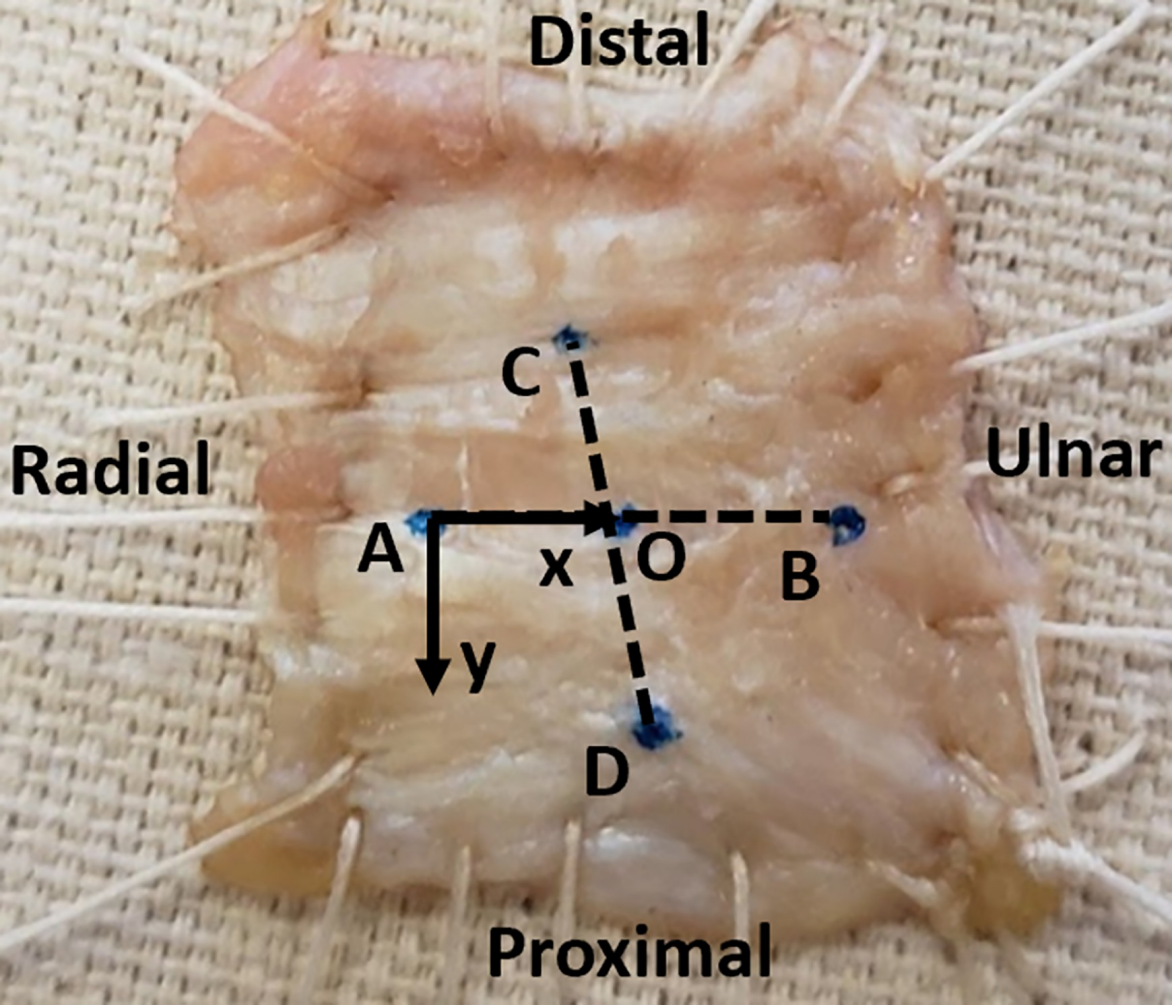
TCL sample preparation. A TCL specimen and a coordinate system associated with the injection point A. Coordinate systems at O, B, C, D are similarly established with x-, y-, z-axes identical, but the origin shifted to these points.

The TCLs of 9 fresh-frozen cadaveric hands were used in this study. IRB approval was not needed because no living human subjects were used in this study, and the data was analyzed anonymously. Tissue volar to the TCL was dissected out, and any thenar and hypothenar muscle insertions were removed from the TCL. The TCL was then removed from the specimen at its boney attachment points and was sutured to a fabric platform. Five injection points were marked on the volar surface of the TCL using tissue marking dye. These points were the center of the TCL (O), and four radial (A), ulnar (B), distal (C), and proximal (D) to the center by 5mm, respectively (Fig 1). The platform was then rigidly fixed in an incubator with the temperature set to 37°C.

### Injection point coordinate system establishment

A coordinate system was established by digitizing each of the five injection points using a MicroScribe Digitizer (MicroScribe GX2, Immersion Corp., San Jose, CA). The x-axis was defined as the AB vector. The z-axis was defined as the cross product of vectors AB and CD. The y-axis was determined by crossing the z-axis with the x-axis. The origin of each coordinate system was placed at each injection point, totaling 5 coordinate systems (Fig 1). The position of each injection point in the robot coordinate system, with the assistance of the MicroScribe to digitize, was determined using the following equation:

$$\left[T_{RX}]=[T_{RT}][T_{TM}][T_{MX}\right]$$ (1)

where [T_RX_] is the transformation matrix between the robot coordinate system and each injection point coordinate system, [T_RT_] is the constant transformation matrix between the robot coordinate system and the coordinate system of the table it is rigidly fixed on, [T_MT_] is the transformation matrix between the MicroScribe coordinate system and the table coordinate system, and [T_MX_] is the transformation matrix between the MicroScribe coordinate system and each injection point coordinate system.

### Footprint coordinate system

**Fig 2:**
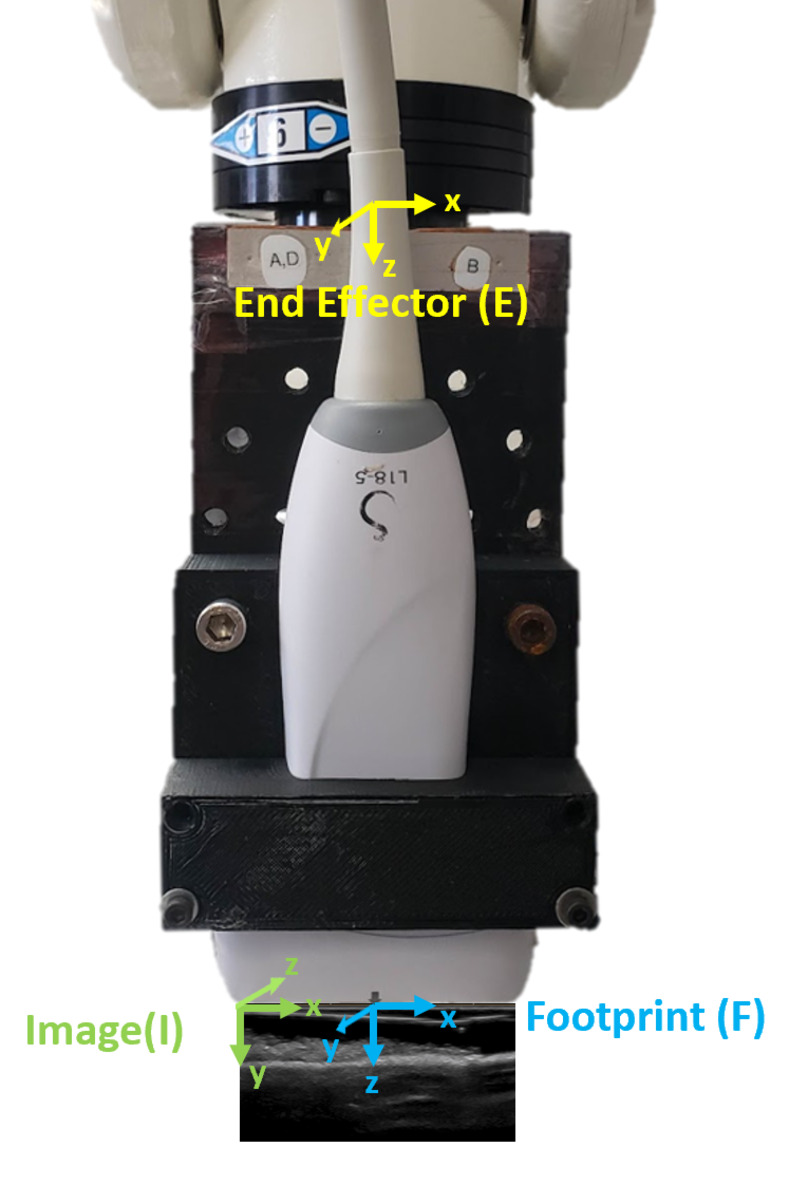
Coordinate systems. The end-effector, image, and footprint coordinate systems rigidly fixed to the end effector of the six-degree of freedom robot arm.

An 18L6 linear array transducer (SuperSonic MACH 30, Hologic Inc., Marlborough, MA, USA) was rigidly fixed to a 6 degree-of-freedom robot using a custom-made probe mount (Fig 2). The image coordinate system of the ultrasound probe is a 3D coordinate system with its origin on the left corner of the probe footprint, which corresponds with the top-left most corner of the ultrasound image. Its x-axis is the horizontal axis of the image, its y-axis is the vertical axis of the image, and its z-axis is the cross product of the y-axis with the x-axis. A footprint coordinate system was established by translating the image coordinate system 26 mm in the x-direction so that its origin was at the center of the probe footprint, and then rotating the image coordinate system 90 degrees about its x-axis. The footprint coordinate system can be represented in the robot coordinate system using the following equation:

$$\left[T_{RF}]=[T_{RE}][T_{EP}][T_{PI}][T_{IF}\right]$$ (2)

where [T_RF_] is the transformation matrix between the robot and probe footprint coordinate systems. [T_RE_] is the transformation matrix between the robot and end effector coordinate systems, which is given by the robot. [T_EP_] and [T_PI_] are the constant transformation matrices between the robot end effector and probe coordinate systems and between the probe and image coordinate systems, respectively, which is pre-calibrated. [T_IF_] is the transformation matrix between the image coordinate system and the footprint coordinate system.

### Injection

CCH with a specific activity ≥125 Collagenase Digestion Units/mg solid (Sigma-Aldrich, St. Louis, MO) was dissolved in phosphate buffered saline to create five solutions with concentrations of 0.4, 0.32, 0.24, 0.16, and 0.08mg/μL so that the units of CCH per 5μL of solution were 50U,100U, 150U, 200U, and 250U, respectively. A microliter syringe with a 30G needle was used to inject 5μL of each solution into one of the five marked injection points at the middle of the TCL thickness.

### Ultrasound scanning procedures

**Fig 3:**
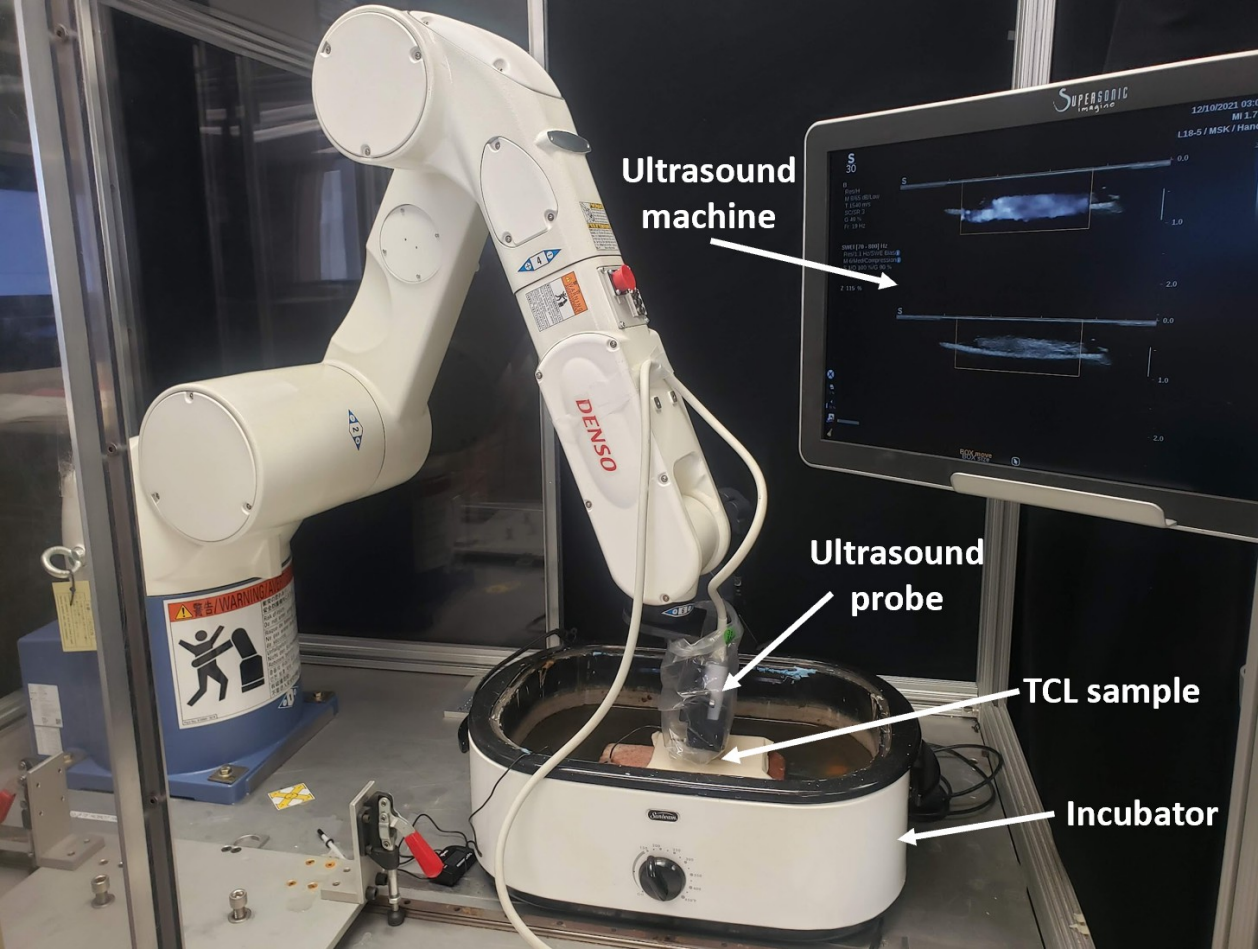
Experimental setup. Experimental setup for scanning procedure.

A thin layer of ultrasound gel was applied to the surface of the TCL, and an ultrasound scan was done of each injection point immediately (0 hour), 2 hours, 4 hours, 6 hours, 8 hours, and 24 hours after injection. For each injection point, the robot was programmed to superimpose the footprint coordinate system with each injection point coordinate system, except that the footprint coordinate system was offset 5 mm in the negative-z direction to allow space for the ultrasound gel. The robot moved the footprint coordinate system to this position using the following equation:

$$\left[T_{RF}]=[T_{RX}][T_{XF}\right]$$ (3)

where [T_RF_] is the transformation matrix between the robot and footprint coordinate system, [T_RX_] is the transformation matrix between the robot coordinate system and each injection point coordinate system, and [T_XF_] is the transformation matrix between each injection point coordinate system and the footprint coordinate system. With the known [T_RF_], the robot is programmed to move [T_RE_] by solving Eq (2). For scanning, the robot translated the probe centered around each injection point 2mm in the positive and 2mm in negative y-directions in the injection point coordinate system in 0.2 mm steps (Fig 3). A shear wave elastography image and B-mode image at each step were captured.

### Shear wave speed measurement

A region of interest for each injection point was defined as a rectangular prism centered on the injection point with a width of x = 1mm, a length of y = 1mm, and a height z that contained the entire TCL thickness. A custom LabVIEW program was used to segment the TCL from each B-mode image based on grayscale value within this region of interest, and the shear wave speed of each TCL point was determined from the corresponding shear wave elastography image. The average shear wave speed measured in the region of interest for each injection point was calculated at the timepoints.

### TCL thickness measurement

TCL thickness was measured from the one B-mode image taken at the injection point. ImageJ was used to measure the distance in the image coordinate system’s y-direction from the most volar point to the most dorsal point of the TCL at the centerline of the ultrasound image.

### Statistical analyses

Changes in shear wave speed and thickness were evaluated using a mixed model repeated measures analysis with time and dose as fixed effects, and sample as a random effect. Pairwise comparisons were determined by applying Tukey’s test. Significance was set to alpha of 0.05. Correlation of shear wave speed and thickness with dose at each timepoint following injection was assessed using Pearson’s product-moment correlation coefficient. All statistical analyses were performed using R, version 4.2.1.

## Results

**Fig 4:**
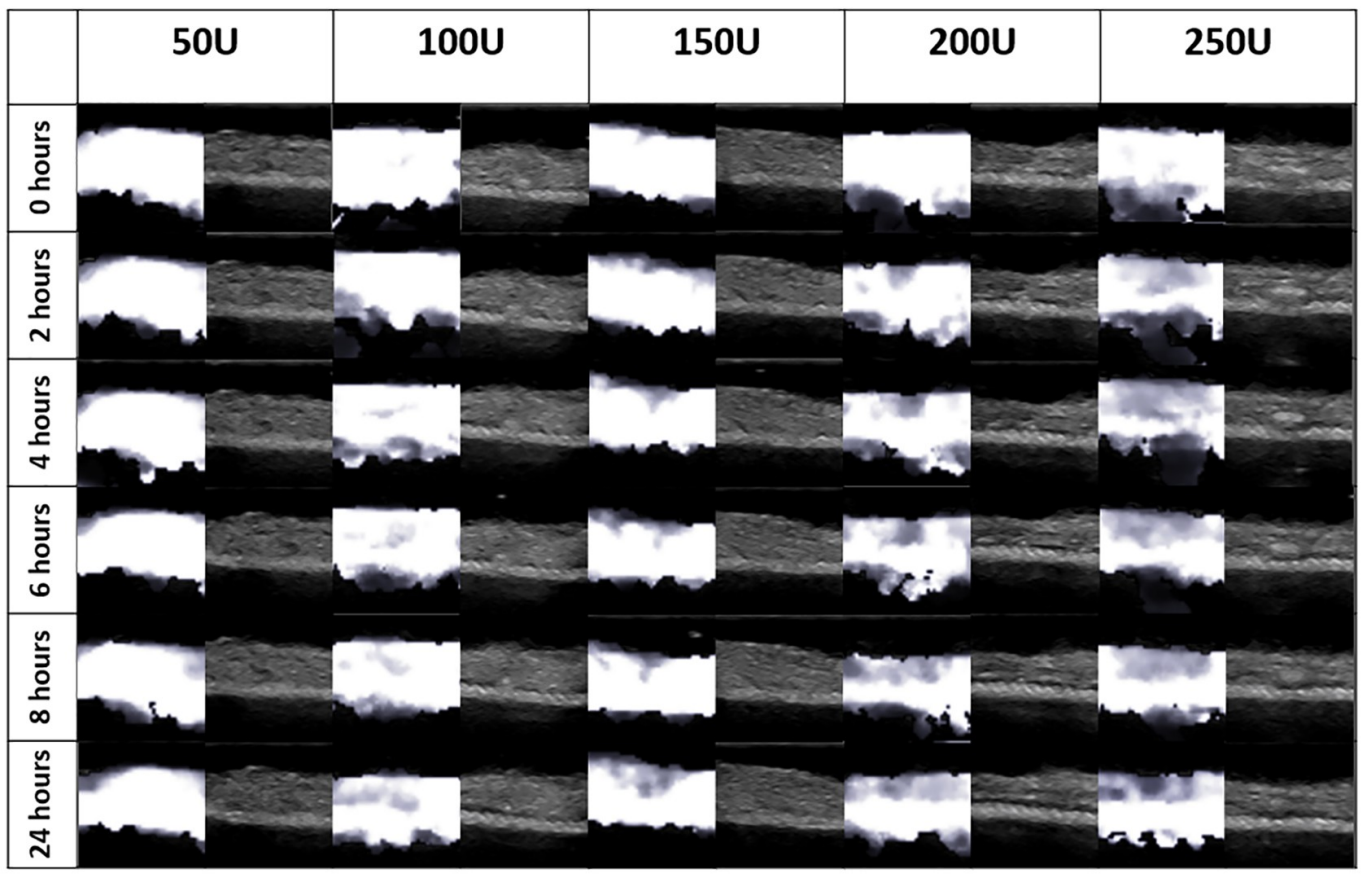
B-mode and shear wave elastography images collected at each injection site and time point for one sample. Greater shear wave speeds are represented by brighter grayscale values in the shear wave elastography images.

For all doses after 24 hours, the TCL was still intact and had no noticeable changes on its outer surface. Shear wave elastography and B-mode images collected at each site of injection for each time point are shown in Fig 4. Slight differences in the texture of the tissue can be detected in the B-mode images after 24 hours. The B-mode images taken at 24 hours after injection show some darker spots scattered throughout the tissue, although this effect is subtle. The shear wave elastography images show the measured shear wave speeds throughout the tissue, with the brighter grayscale values corresponding to higher shear wave speeds. Little change is seen overtime in the shear wave elastography images for doses of 50U and 100U. For doses of 150U, 200U, and 250U of CCH, the shear wave speeds can be seen first decreasing at the site of injection, and then this effect expanding outwards over time.

**Fig 5:**
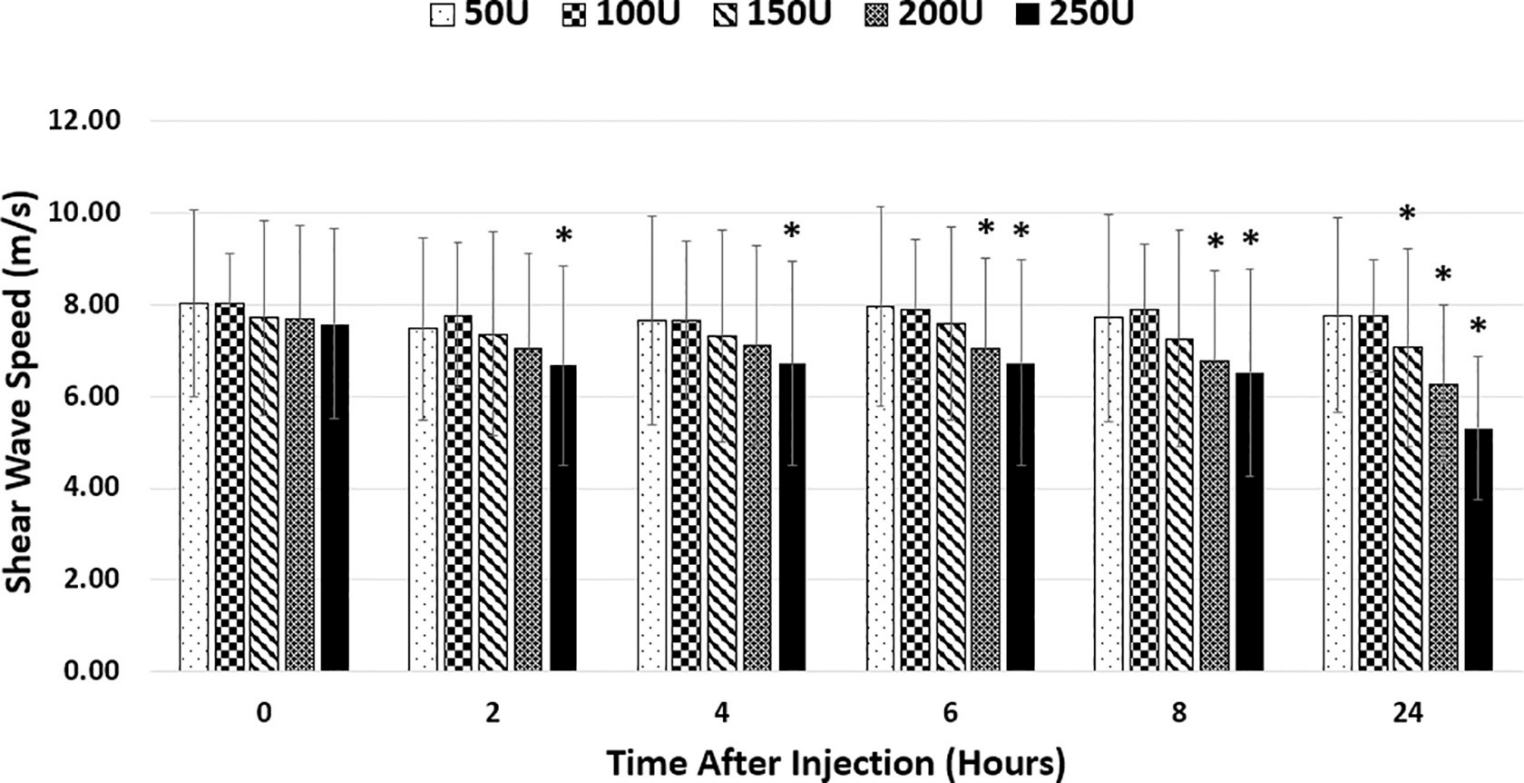
Shear wave speed results. Shear wave speed of individual doses at different timepoints. Significant change in shear wave speed from time 0 within dose signified by (*).

Correlation coefficients between shear wave speed and dose were -0.87, -0.97, -0.98, -0.94, and -0.96 for 2, 4, 6, 8, and 24 hours following injection, respectively. Further comparison showed that there were no statistical differences in shear wave speed at time zero across the five doses (p = 0.963) (Fig 5). There were no significant changes in shear wave speeds in any of the following timepoints within the doses of 50U (p = 0.866) or 100U (p = 0.880). For the dose of 150U, shear wave speeds decreased after 24 hours from 7.71±2.13 m/s to 7.06±2.16, for an average decrease of 8.35% (p = 0.041). For the dose of 200U, shear wave speeds decreased from 7.70±2.03 m/s at time zero to 7.05±1.97 m/s at 6 hours (p = 0.041), 6.77±1.96 m/s at 8 hours (p = 0.007), and 6.26±1.74 m/s at 24 hours (p<0.001), an average decrease of 8.5%, 12.05%, and 18.70%, respectively. For the dose of 250U, shear wave speed decreased at every following timepoint compared to that at time zero. Shear wave speeds decreased from 7.58±2.06 m/s at time zero to 6.68±2.17 m/s (p = 0.002), 6.71±2.23 m/s (p = 0.008), 6.73±2.23 (p = 0.004), 6.53±2.26 (p = 0.002), and 5.31±1.57 m/s (p<0.001), at 2, 4, 6, 8, and 24 hours, respectively. These equated to decreases of 11.96%, 11.46%, 11.20%, 13.92% and 30.01% at 2, 4, 6, 8, and 24 hours, respectively.

**Fig 6:**
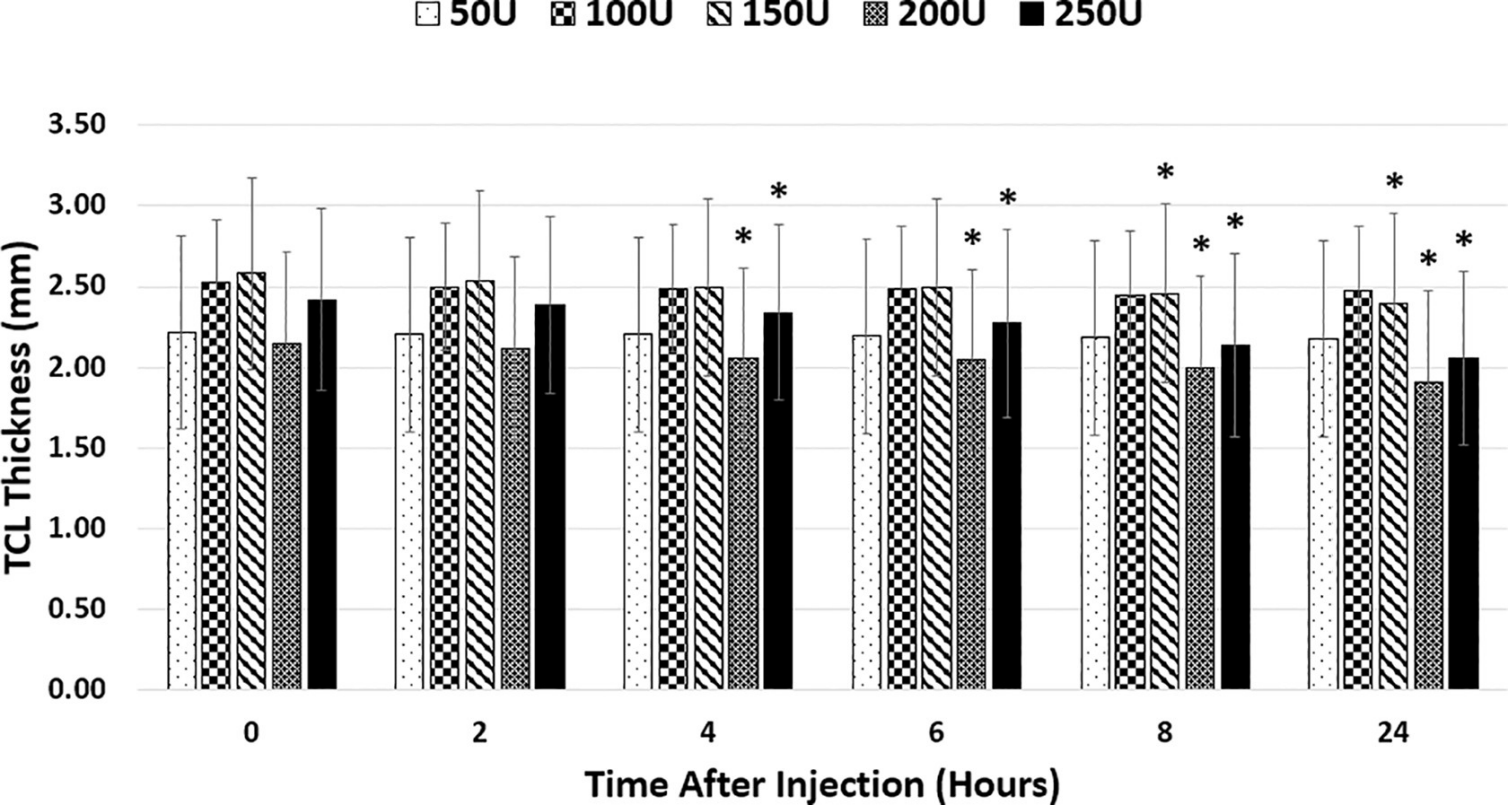
Thickness results. TCL thickness of individual doses at different timepoints. Significant change in thickness from time 0 within dose signified by (*).

Correlation coefficients between thickness and dose were -0.01, -0.13, -0.23, -0.42, and -0.54 for 2, 4, 6, 8, and 24 hours following injection, respectively. TCL thicknesses for the 50U, 100U, 150U, 200U, and 250U doses were 2.22±0.6 mm, 2.52±0.39 mm, 2.58±0.59 mm, 2.15±0.57 mm, and 2.42±0.56 mm, respectively. No significant difference in thickness was found between CCH doses at time 0. TCL thickness for the 50U and 100U doses did not significantly change over time (Fig 6). For the 150U dose, TCL thickness significantly decreased after 8 hours to 2.46±0.55 mm (p = 0.004) and after 24 hours to 2.39±0.56 mm (p = 0.045), an average decrease of 4.74% and 7.28%, respectively. For the 200U dose, TCL thickness significantly decreased after 4 hours to 2.05±0.56 mm (p = 0.003), after 6 hours to 2.04±0.56 mm (p = 0.011), after 8 hours to 2.00±0.56 mm (p = 0.028), and after 24 hours to 1.91±0.56 mm (p = 0.009), an average decrease of 6.78% and 10.97%, respectively. For the 250U dose, TCL thickness significantly decreased after 4, 6, 8, and 24 hours to 2.34±0.54 mm (p = 0.015), 2.27±0.58 mm (p<0.001), 2.14±0.57 mm (p<0.001), and 2.06±0.54 mm (p<0.001), respectively.

## Discussion

The TCL is constituted of collagen fibers types I and III and is relevant to carpal tunnel syndrome due to its volar restriction of the median nerve. The most common surgical procedure is to transect the TCL, disrupting the anatomy of the TCL and resulting in side effects, such as pillar pain and hand weakness. Biochemically degrading the TCL through CCH injection may provide an alternative to carpal tunnel release surgery, but the effects of CCH on TCL tissue structure have not yet been explored. Breaking down the collagen fibers in the TCL could decrease TCL stiffness, allowing the TCL to palmarly bow and create more space in the carpal tunnel for the median nerve. Elastic modulus and thickness are two major factors that affect stiffness, and lowering these properties would lower stiffness in the TCL. As the first steps, we assessed the dose- and time-dependent effects of Collagenase Clostridium Histolyticum on TCL elastic modulus and thickness using isolated TCL samples from cadaveric hands.

Robot-assistance allowed for precise and accurate positioning of the ultrasound probe, ensuring that the same regions of interest were imaged at every timepoint. Shear wave elastography ultrasound imaging and B-mode ultrasound imaging were used in this study to assess the mechanical and morphological parameters of the TCL. B-mode ultrasound imaging was used to measure TCL thickness, a method that has previously been shown to be accurate with a standard error of 0.02–0.07mm [20]. Shear wave elastography measures the speed of shear waves traveling through the tissue being imaged and has previously been used to assess TCL stiffness in pianists [23]. Elastic modulus can be determined by multiplying shear wave speed with the density of the tissue being imaged. Although elastic modulus was not directly determined in this study, changes in elastic modulus can be represented by changes in shear wave speed.

Strong negative correlation was found between shear wave speed and dose at every timepoint after injection. This suggests that dosage does affect TCL degradation, and these effects can be seen as soon as 2 hours following injection. Correlation analysis between thickness and dose revealed weak negative correlation at 2, 4, and 6 hours following injection, and moderate negative correlation at 8 and 24 hours following injection. The correlation between thickness and dose was weaker than the correlation between shear wave speed and dose but became stronger as time increased. These results showing that CCH injection has a stronger effect on TCL shear wave speed than thickness suggests that TCL mechanical property (i.e., elastic modulus) is more sensitive to CCH injection than geometric measures.

For injection of doses of 100U or fewer, the enzymatic activity is not apparent, as there were no significant changes in shear wave speed at any timepoints. As the dose increases to 150U and above, the tissue breaks down, as indicated by decrease in shear wave speed. For example, 250U of CCH decreased the shear wave speed by >10% after two hours and >30% after 24 hours compared. Similarly, TCL thickness decreased 11.75% and 14.92% at 8 and 24 hours, respectively. Although TCL thickness may not play as important of a role as elastic modulus in allowing the TCL to palmarly bow and decompress the median nerve, previous studies have associated greater TCL thickness with carpal tunnel syndrome. Marquardt et al. found that TCL thickness in patients with carpal tunnel syndrome was 30.9% greater in the central region of the TCL that that in healthy subjects [7]. Villar et al. found that within patients with carpal tunnel syndrome, greater TCL thickness can be associated with greater severity of symptoms [24].

None of the doses of CCH resulted in high enough degradation to visibly change the outer surface of the TCL or break through the entire TCL thickness and create holes, suggesting that the effects of the CCH were contained within the boundaries of the TCL. Too much degradation not contained within the boundaries of the TCL could affect the biomechanical function of the TCL or allow the CCH to leak out of the TCL and affect the surrounding structures, such as the thenar muscles or flexor tendons. The 250U dose of CCH resulted in the greatest decrease in shear wave speed and thickness over 24 hours and took the shortest amount of time to decrease shear wave speed and thickness. Increasing the dose greater than 250U for one injection may be difficult as 250U/5μL was the highest dose able to be prepared, and the amount of solution able to be injected into one area without leaking was limited to a small amount, such as 5μL.

There were several limitations to this study. Injection points were manually marked on the TCL using blue dye and digitized using a MicroScribe digitizer. Human error in needle placement or digitization would result in the region of interest not being centered on the true injection point and could impact the results. Also, variation in tissue density could impact how the solution spread within the TCL after injection, causing it to not be centered within the region of interest. Because this study was performed *in vitro*, effects of the body could not be taken into account, and these results may not accurately represent how the TCL would react to CCH injection as a treatment for carpal tunnel syndrome *in vivo*.

This study found that higher doses of CCH result in greater and faster degradation of the TCL. Furthermore, injecting 100U or less of CCH has no significant impact on the mechanical properties of the TCL. Injecting 150U or 200U of CCH decreased TCL elastic modulus and thickness after a prolonged time (i.e., greater than 8 hours). A dose of 250U was the most effective for TCL degradation, significantly decreasing elastic modulus after 2 hours and thickness after 4 hours, as well as having the greatest percent decrease in elastic modulus and thickness after 24 hours. These results can aid in selecting a dosage for effective manipulation of TCL structure *in situ*. Also, our methods of using ultrasound imaging for thickness and shear wave speed measurements can be applied *in situ* and *in vivo*. The effects of each injection were examined in a 1×1mm area of interest, but in order to have a significant impact on overall TCL elastic modulus and thickness to increase carpal tunnel space, multiple injections of CCH would be needed. Future studies will investigate the effect of multiple injections of 250U of CCH on carpal arch morphology to determine the ideal injection configuration to decompress the median nerve.

## Conclusions

This study assessed the effects of CCH injection on TCL stiffness *in vitro* as a first step in investigating the potential of CCH injection as a treatment for carpal tunnel syndrome. Shear wave elastography and B-mode ultrasound imaging were used to assess TCL elastic modulus and thickness, respectively. Robot assistance allowed for precise positioning of the ultrasound probe at each timepoint. Strong negative correlation was found between shear wave speed and dose, but the correlation between thickness and dose was weaker, indicating that TCL shear wave speed may be more affected by CCH injection than TCL thickness. Injecting 150U or greater of CCH to the TCL resulted in decreased TCL elastic modulus and thickness at 24 hours, with the 250U dose resulting in the greatest decrease in shear wave speed and thickness. Doses of 100U or fewer had no significant effect on TCL shear wave speed or thickness, suggesting that higher doses may be needed for TCL degradation. Future studies will assess the effects of multiple CCH injections on the TCL *in situ* and may use these findings as a guide for determining dosage.

## Supporting information

S1 Dataset Raw data. The raw data points for shear wave speed and thickness used for statistical analysis.
(XLSX)

S1 Fig Sample ultrasound image. A shear wave elastography (top) and B-mode (bottom) ultrasound image of the TCL sample used in the study.
(TIF)
