# Supplementary figures and images for: Dose- and time-dependent effects of collagenase clostridium histolyticum injection on transverse carpal ligament elastic modulus and thickness in vitro
Source: PLoS One. 2022 Dec 1;17(12):e0277187. doi: 10.1371/journal.pone.0277187 (PMC9714872; doi:10.1371/journal.pone.0277187)

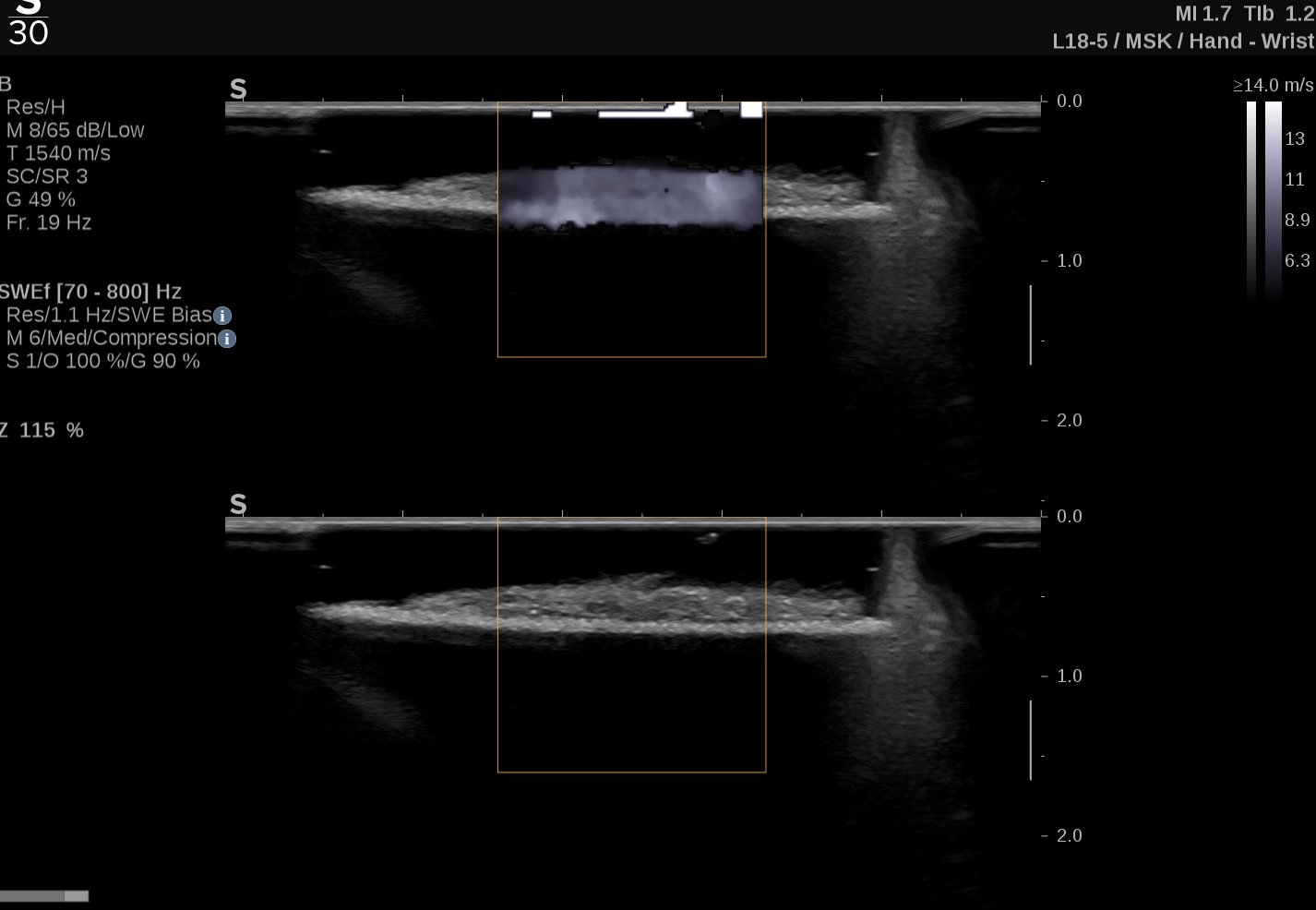

Supplement: S1 Fig — A shear wave elastography (top) and B-mode (bottom) ultrasound image of the TCL sample used in the study. (TIF) [file pone.0277187.s002.tif]
